# An adjustable acoustic metamaterial cell using a magnetic membrane for tunable resonance

**DOI:** 10.1038/s41598-024-65819-2

**Published:** 2024-07-01

**Authors:** Alicia Gardiner, Roger Domingo-Roca, James F. C. Windmill, Andrew Feeney

**Affiliations:** 1 Centre for Medical and Industrial Ultrasonics, James Watt School of Engineering, University of Glasgow, Glasgow, G12 8QQ UK; 2 Centre for Ultrasonic Engineering, Department of Electronic & Electrical Engineering, University of Strathclyde, Glasgow, G1 1XW UK

**Keywords:** Acoustic metamaterials, Magnetic membranes, Resonance tunability, Stereolithography printing, Superparamagnetism, Polymers, Mechanical engineering, Photoacoustics, Acoustics

## Abstract

Acoustic metamaterials are growing in popularity for sound applications including noise control. Despite this, there remain significant challenges associated with the fabrication of these materials for the sub-100 Hz regime, because acoustic metamaterials for such frequencies typically require sub-mm scale features to control sound waves. Advances in additive manufacturing technologies have provided practical methods for rapid fabrication of acoustic metamaterials. However, there is a relatively high sensitivity of their resonant characteristics to sub-mm deviations in geometry, pushing the limits of additive manufacturing. One way of overcoming this is via active control of device resonance. Here, an acoustic metamaterial cell with adjustable resonance is demonstrated for the sub-100 Hz regime. A functionally superparamagnetic membrane—devised to facilitate the fabrication process by eliminating magnetic poling requirements—is engineered using stereolithography, and its mechanical and acoustic properties are experimentally measured using laser Doppler vibrometry and electret microphone testing, with a mathematical model developed to predict the cell response. It is demonstrated that an adjustable magnetic acoustic metamaterial can be fabricated at ultra-subwavelength dimensions ($\le \lambda$/77.5), exhibiting adjustable resonance from 88.73 to 86.63 Hz. It is anticipated that this research will drive new innovations in adjustable metamaterials, including wider frequency ranges.

## Introduction

Acoustic metamaterials (AMMs) are structures with acoustic properties enhanced or solely defined by their geometry, rather than by their intrinsic mechanical properties^1^. These are, typically, artificial structures of periodic, identical units with feature dimensions within the order of magnitude of the operational wavelength, where phononic crystals are a prime example^2^. Within the last 20 years, the rate of progress of AMM research has exponentially increased, and the field now demonstrates substantial potential for real-world applications, such as architectural noise control^3,4^ and diagnostic ultrasonic imaging^5,6^. Despite this, AMMs have yet to be widely implemented in industrial applications. A major factor is the limitation of current manufacturing methods to deliver the structural geometries required for their operation at certain frequencies. A principal challenge for the fabrication of miniature, low-frequency (20–500 Hz) acoustic devices based on metamaterials is the requirement for relatively small feature sizes and complex geometries. The size of acoustic metamaterials for this frequency range can be in the order of mm, which poses a significant challenge for fabrication tolerances. Conventional techniques such as milling, casting, and injection moulding are traditional options for the fabrication of AMMs^7,8^, but the required resolution is difficult to ensure with these methods. Additive manufacturing is a promising candidate to support the fabrication of AMMs^9^. Additive manufacturing techniques can be broadly separated into three categories, comprising vat polymerisation, powder bed fusion, and extrusion/deposition. Stereolithography apparatus (SLA) 3D-printing has been identified as a highly suitable method of AMM fabrication^10^ given its layer-by-layer fabrication mechanism, where 2D cross-sections are cured with a targeted photopolymerisation mask of ultraviolet or visible light which is applied in successive cycles to build a 3D structure^11^.

A principal challenge for the fabrication of AMMs at the sub-mm scales required for low frequency applications arises from the achievable accuracy in the additive manufacturing of the metamaterial cells. Sub-mm geometries are required, but it is not always possible to ensure the accuracy of a 3D-printed specimen given the limits of available SLA techniques (and even if SLA 3D-printing can be used to fabricate structures with layer thicknesses of the order of 25 μm^12,13^, well within the microscale). One way this could be overcome is through adjustable AMMs, those where the dynamic or resonant properties can be precisely adjusted and modulated post-fabrication to deliver the desired response. Key properties of interest for AMMs are resonance frequency, bandwidth, and attenuation. There are multiple examples in the literature regarding tuning mechanisms for AMMs^14^. An adjustable flexural wave cloak successfully operated within 900–1200 Hz and was comprised of concentric layers of polydimethylsiloxane and piezoelectric patches—exploiting the piezoelectric effect for the active mechanism^15^. A mechanical system converts the topological states of a metamaterial using motors, thereby producing extraordinary vibrational behaviours^16^. A different mechanical approach for acoustic modulation is demonstrated by using an array of pneumatically-actuated Helmholtz resonators to adjust the operational bandwidth at low frequencies^17^. Another example of a Helmholtz resonator array, with water as the transmission medium, used temperature to change acoustic bandgap properties^18^. In another example of an adjustable AMM, electromagnets were used to apply mechanical stress to a membrane via magnetic clamps, realising a device capable of a 40% resonant bandgap shift^19^. A unit cell with a piezoelectric diaphragm was developed^20^, with closed-loop control^21^, and a broad tunable effective density, resulting in modifiable acoustic properties. Another example consisting of a hydrogel composite was demonstrated with the capacity to tune its acoustic impedance via fillable channels, dependent on the filler material^22^. Similarly, an ink-jet 3D-printed soft mechanical metamaterial was successfully shown to change its effective mechanical properties depending on hydration levels^23^, resulting in tunable stress-strain curves and change its isotropic/anisotropic features as desired. An example of a magnetic membrane used in an AMM has also been used to demonstrate tunable effective density over a wide frequency bandwidth^24^.

The current knowledge gap, and the novelty that this research addresses, is that none of the competing technologies have been shown to be exclusively fabricated through additive manufacturing, nor have they been used to demonstrate adjustable resonance in the sub-100 Hz regime. The core aim of this research is to demonstrate the additive manufacture of an AMM through SLA 3D-printing, which displays adjustable resonance via magnetic actuation. This is achieved using an AMM cell based on a Helmholtz resonator coupled to a magnetic membrane, where the 3D-printed prototype is displayed in Fig. 5. The device’s membrane is comprised of a 3D-printed functionally superparamagnetic resin. Under an applied magnetic field, instantaneous force is applied to the membrane, resulting in increased tension and therefore actively modifying its resonance frequency. The function of the magnetic membrane allows for pre-stress to be applied instantaneously and without any applied physical contact. The significance of being able to entirely 3D-print an adjustable AMM is that this will make possible the production of functional structures, feature sizes and geometries necessary to deliver a new generation of adaptable devices for low frequency applications.

## Results

### Resonance frequency of the membrane

**Figure 1:**
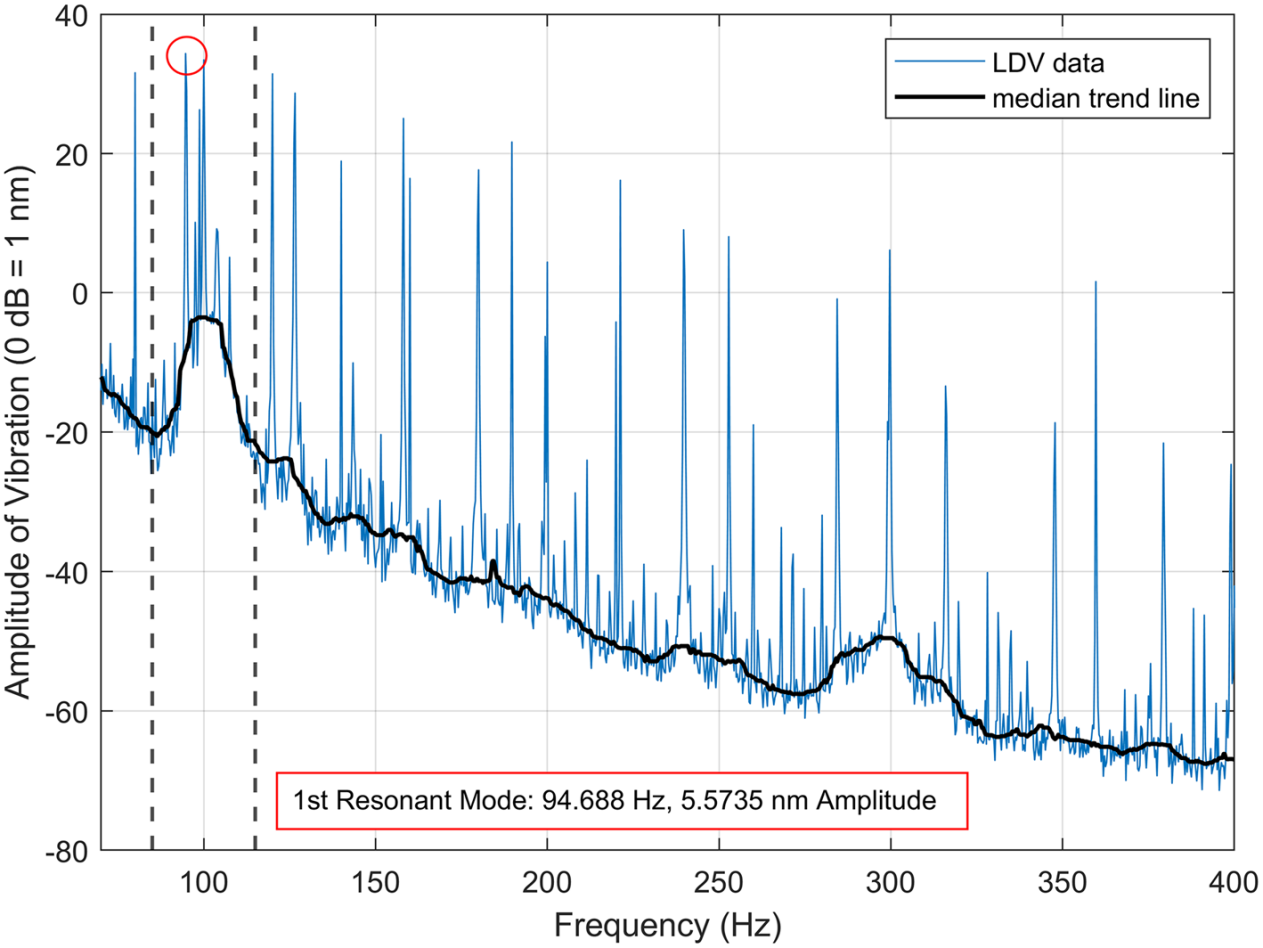
Measured vibration (using laser Doppler vibrometry) of the clamped magnetic membrane. The solid blue line displays the raw data, and the solid black line shows the median trend line (window size, 40). The resonant peak is marked via the red annotation.

The first resonant mode of the magnetic membrane at 94.96 Hz is shown in Fig. 1. The corresponding mechanical pre-stress was calculated to be 3.187 N/m using the mathematical model (see Equation S1).

### Mechanical and magnetic properties of the 3D-printable resin

**Figure 2:**
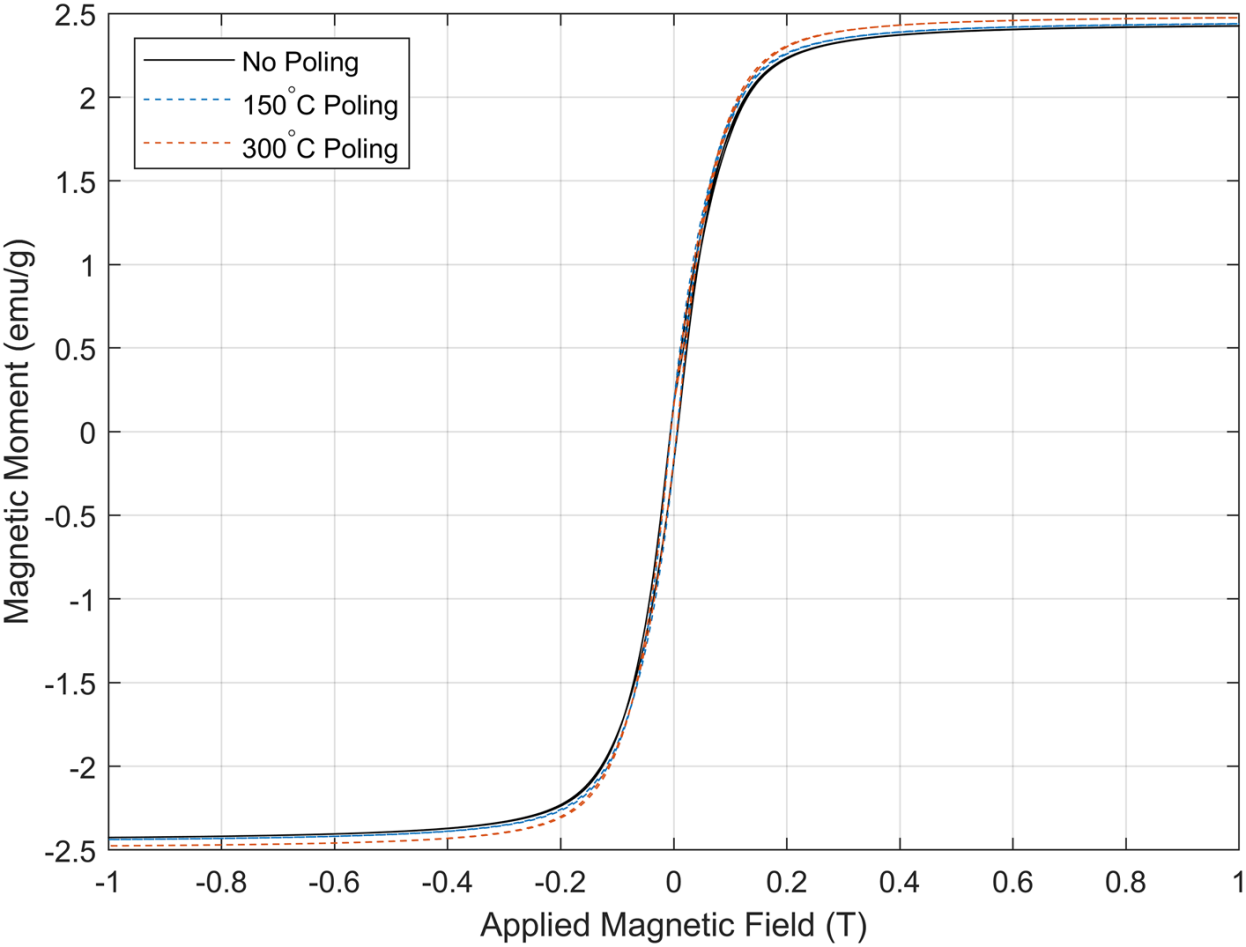
Magnetic hysteresis loop (obtained via Superconducting Quantum Interference Device magnetometer) of the custom-made, SLA 3D-printable resin. The solid black line shows the non-poled sample, and the blue and orange dashed lines show, respectively, the samples poled at 150 °C and 300 °C.

The Young’s modulus, *E*, was determined by placing the thickness of the magnetic membrane (*h* = 260 μm), the membrane radius (*R* = 13 mm), and the frequency of the first membrane mode ($f_1$ = 94.69 Hz, determined experimentally) into Equation S2^25^, resulting to 17.89 MPa. The density was measured to be 1185.23 kg/m^3^, and $\nu$ was estimated to be 0.35 based on the reported values in the literature of similar materials—see Supplementary Information. Three samples were post-processed with different poling regimes (no poling, 150 °C, and 300 °C), demonstrating the functionally superparamagnetic properties of the material. The measured magnetisation per gram as a function of the applied magnetic field is displayed in Fig. 2. The magnetic properties derived from this data are shown in Table 1, and further details are displayed in Supplementary Table S2.

**Table 1:** Magnetic properties of the membrane material developed for this acoustic metamaterial cell, showing the effect of different poling regimes on the bespoke magnetic resin.

Magnetic properties of resin under different poling regimes.			
Poling regime	Magnetisation of saturation (emu/g)	Saturation remanence (emu/g)	Magnetic coercivity (mT)
None	2.4265	0.1660	4.9915
150 °C, 2 h	2.4389	0.1726	4.9964
300 °C, 2 h	2.4753	0.1502	4.9927

These results confirm the effectively superparamagnetic behaviour of the resin, as the poling regime has no significant effect on the magnetic properties of the resin at the given nanoparticle concentrations. Figure 2 shows minimal change in the magnetisation regardless of the poling regime, and hence the resin can be considered to be at maximal magnetisation of saturation without any poling. A magnetisation of saturation of 2.4 emu/g can be assumed for the mathematical model.

A marginal increase in saturation remanence and coercivity was observed in the 150 °C poling regime with respect to the non-poled sample. Nevertheless, the sample poled at 300 °C displayed lower values than those measured from the sample poled at 150 °C. For perfect superparamagnetic behaviour, the saturation remanence and magnetic coercivity would be zero, while Table 1 displays small, non-zero values of these properties for all poling regimes, characteristic of a very soft ferromagnet. This may be due to the aggregation of a small number of magnetite nanoparticles, forming clusters that behave like larger (micro)particles that result in ferromagnetic behaviour instead of superparamagnetic. With a coercivity of around 5 mT, the material will orientate its magnetic response to the direction of externally applied magnetic fields, therefore removing the need for magnetic poling. Similarly, the saturation remanence is too small to have an impact on the applied magnetic field. For these reasons, the resin can be considered effectively superparamagnetic.

### Comparison of mathematical model to experiment

#### Resonant behaviour

**Figure 3:**
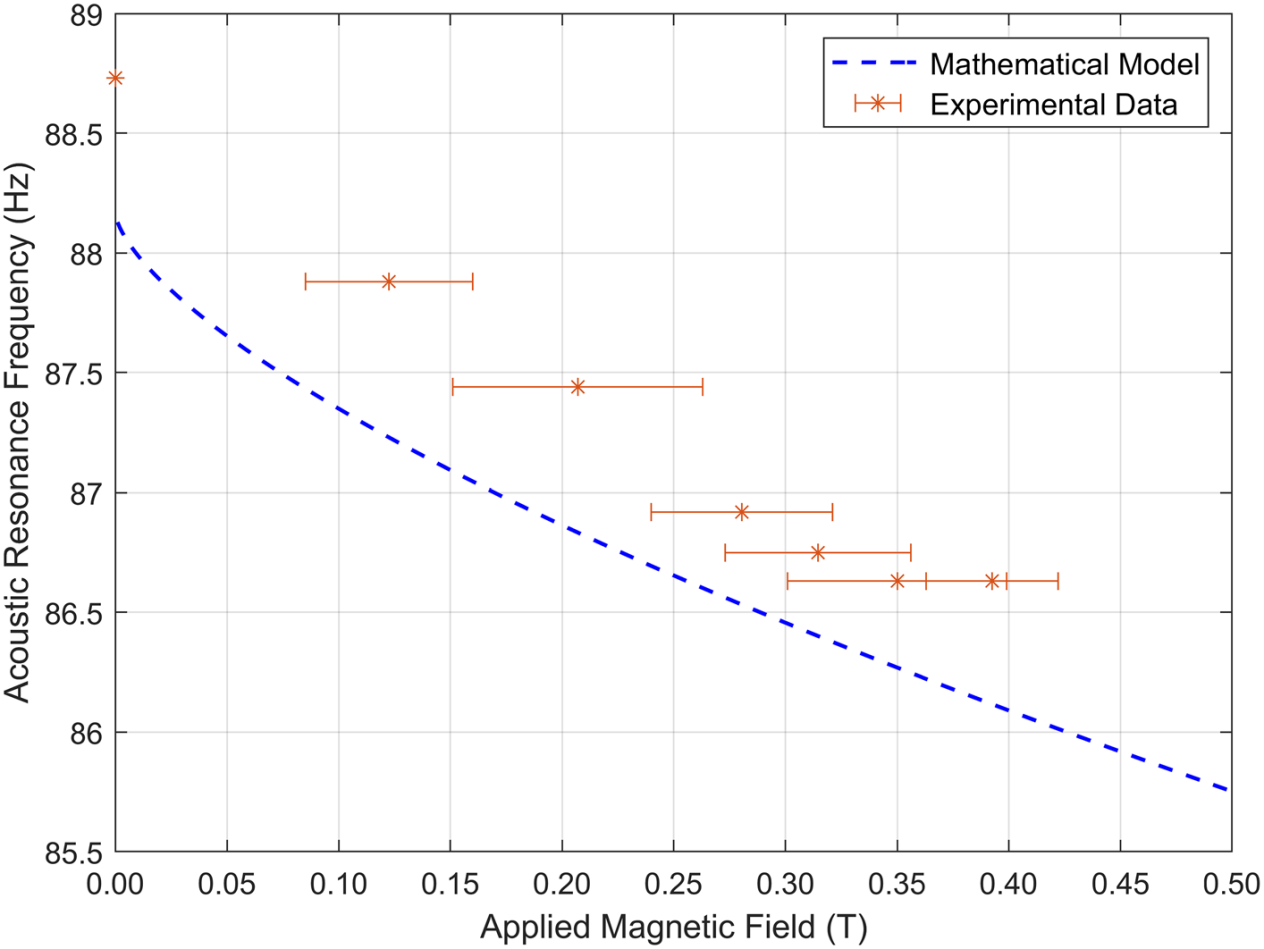
Acoustic resonance frequency as a function of applied magnetic field, for both experimental (red stars) and mathematical modelling approaches (dashed blue line). Error bars show recorded magnetic field bounds for each regime—see Supplementary Table S3.

The adjustable AMM design was excited over a bandwidth of 50–150 Hz, with the resulting fundamental resonant mode recorded under different magnetic field regimes (Supplementary Table S3). The experimental results are plotted alongside the theoretical model, as shown in Fig. 3, with the membrane pre-stress, and the measured mechanical and magnetic properties used to inform the mathematical model. The recorded resonance frequencies are between 88.73 Hz (at 0 mT) and 86.63 Hz (at 392.5 mT), with steady intervals of magnetic field data displaying a trend in agreement with the mathematical approximation. The adjustable bandwidth of the experimental device is 2.10 Hz at a peak applied magnetic field of 392.5 mT. The mathematical model exhibits an expected adjustable bandwidth range of 1.90–2.11 Hz under the same magnetic field band, demonstrating a highly accurate estimate of device performance. The predicted resonance frequency without an applied magnetic field is 88.18 Hz, differing by 0.55 Hz from the experimental results.

Within the dataset presented in Fig. 3, the recorded resonance frequencies for applied magnetic fields of 350 mT and 392.5 mT are observed to be similar to one another. This is arributed to the smaller additive increase in magnetic field as more magnets are applied—see Supplementary Fig. S2 for further detail. The resonance frequency of the AMM cell is instantaneously tunable under an externally applied magnetic field, showing great agreement between the experimental data and the proposed mathematical model, therefore introducing a new type of adjustable AMM into the field of experimentally viable metamaterials.

#### Effect of applied magnetic field on signal power increase at resonance

**Figure 4:**
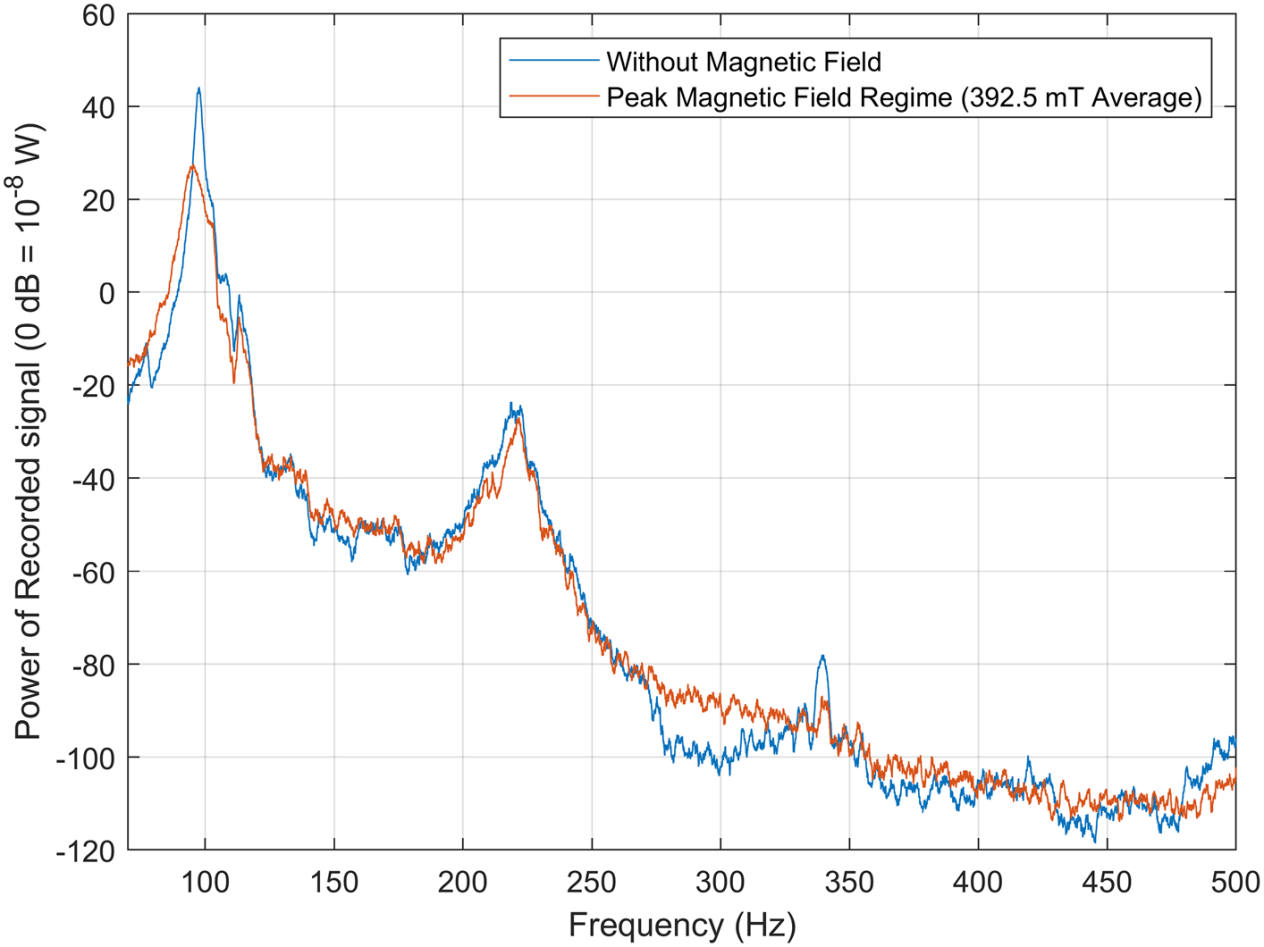
Acoustic power spectra of the acoustic metamaterial cell obtained using an electret microphone for the cases of no applied external magnetic field (0 mT, blue line), and peak magnetic field regime (392.5 mT average, orange line). The signals were recorded over a bandwidth of 70–500 Hz.

Bandwidth was recorded at zero applied magnetic field (0 mT) and maximum applied magnetic field (392.5 mT), in order to compare the spectral power at resonance. Figure 4 shows the results for 70 Hz to 500 Hz, with 2 s per Hz (25,000 data points per second). The signal power is displayed in dB with a reference power of $10^{-8}$ W. The peak signal power at resonance without an applied magnetic field is 13.51 dB higher than that with maximum applied magnetic field (392.5 mT). The signal with and without a magnetic field spans 144.5 dB and 162.7 dB from resonance to non-resonance, respectively.

## Discussion

This work reports the design, mathematical modelling, fabrication, and testing of an adjustable acoustic metamaterial cell for sub-100 Hz operation, that is entirely 3D-printed. This is achieved by coupling a Helmholtz resonator to a magnetic membrane, such that the tension on the membrane is controlled via application of an external magnetic field. The device has successfully shown change of its resonant behaviour under applied magnetic fields, and reveals strong agreement with the proposed mathematical model. Despite the narrow tunable bandwidth, this device is competitive with regards to the literature. To contrast with current research, two magnetic membrane examples from the literature show a minimum resonance frequency of 1200 Hz^26^ and 240 Hz^24^ respectively, well above the sub-100 Hz regime explored in the proposed device. Furthermore, these membrane examples employ more specialist fabrication techniques with custom equipment, such as screen-printing and mould curing, and have not been integrated into an acoustic device. An example that explores acoustic tunability without membranes involves an array of Helmholtz resonators with a pneumatic adjustment mechanism^17^. This design requires complex control hardware to operate, and displays very little precision in resonance tunability. It offers 8–20 dB noise mitigation exclusively in frequencies above 100 Hz, unable to operate in the sub-100 Hz regime. Another tunable metamaterial utilizing Helmholtz resonators with embedded necks was shown to exhibit tunable sound absorption across a 99 Hz bandwidth^27^, however for a minimum frequency of 197 Hz and where tuning across the narrow frequency bands, like in this work, was not demonstrated. The approach presented in this research shows potential for highly precise tuning of device response. Therefore, for low-frequency ultra-subwavelength devices, such as the one presented in this work (88.73–86.63 Hz, and $\lambda$/77.5, respectively), an adjustable bandwidth of 2.1 Hz with 162.7 dB increase in signal power at resonance is significant.

The mathematical model developed to describe the acoustic behaviour of the proposed AMM cell consists of a 3 degrees of freedom (DOF) mass-spring-damper system. Adding further elements would increase the complexity, with each additional DOF adding an order of magnitude to the required computation. When analysing the mathematical model, it was observed that *E* greatly informs the gradient of the plot of Fig. 3. The acoustic resonance frequency difference between the predicted and experimental models is attributed to minor variations in the membrane pre-stress over time. As it can be observed from Fig. 3, the gradient of the experimental data is largely in line with the mathematical prediction. This could be because *E* is an intrinsic value of the material and is nominally fixed after fabrication, whereas membrane tension is more susceptible to fluctuation over time. Identifying *E* as a parameter proportional to the gradient of the predicted behaviour of the AMM cell is a key observation when attempting to improve the bandwidth. By examining Eqs. (4–6), important factors to increasing the magnetic-based applied tension (and, therefore, the sensitivity of the bandwidth mechanism) are the magnetisation, thickness, and Young’s modulus of the membrane.

The experimental testing recorded a frequency modulation between 88.73 Hz and 86.63 Hz at 0 mT and 392.5 mT applied magnetic field, respectively. The total bandwidth change of the device was recorded to be of 2.1 Hz at 392.5 mT, which was confirmed by the proposed mathematical model (which provides a predicted value of 1.90–2.11 Hz). The resonance frequency in the absence of magnetic field was measured to diverge, only by 0.55 Hz from that predicted by the mathematical model. It was observed that modest changes in the membrane pre-stress significantly changed the initial non-magnetic resonance frequency (*y*-intercept). The influence of the fit of the membrane clamp on the recorded data is to be expected. There are inevitable minor variations in geometrical dimensions and material properties leading to minor discrepancies between modelling and experimental observations. Similarly, there are interfering factors to consider, such as signal distortion and background noise, that are difficult to eliminate and may corrupt data readings. Minor variations in membrane pre-stress, and the metrological methods applied to define it, have resulted in a marginal discrepancy between the predicted and experimental frequency responses. When applying a magnetic field, it was observed that the acoustic resonance at 350 mT does not significantly differ from that measured at 392.5 mT. This is attributed to the fact that, at this stage, the marginal increase in magnetic field is not capable of inducing any extra degree of tension on the membrane, therefore reaching a steady state for the acoustic resonance of the AMM cell. The difference between applied magnetic fields for each successive magnet regime is displayed in Supplementary Fig. S2, with a steep drop in field increase observed after 3 magnets.

**Figure 5:**
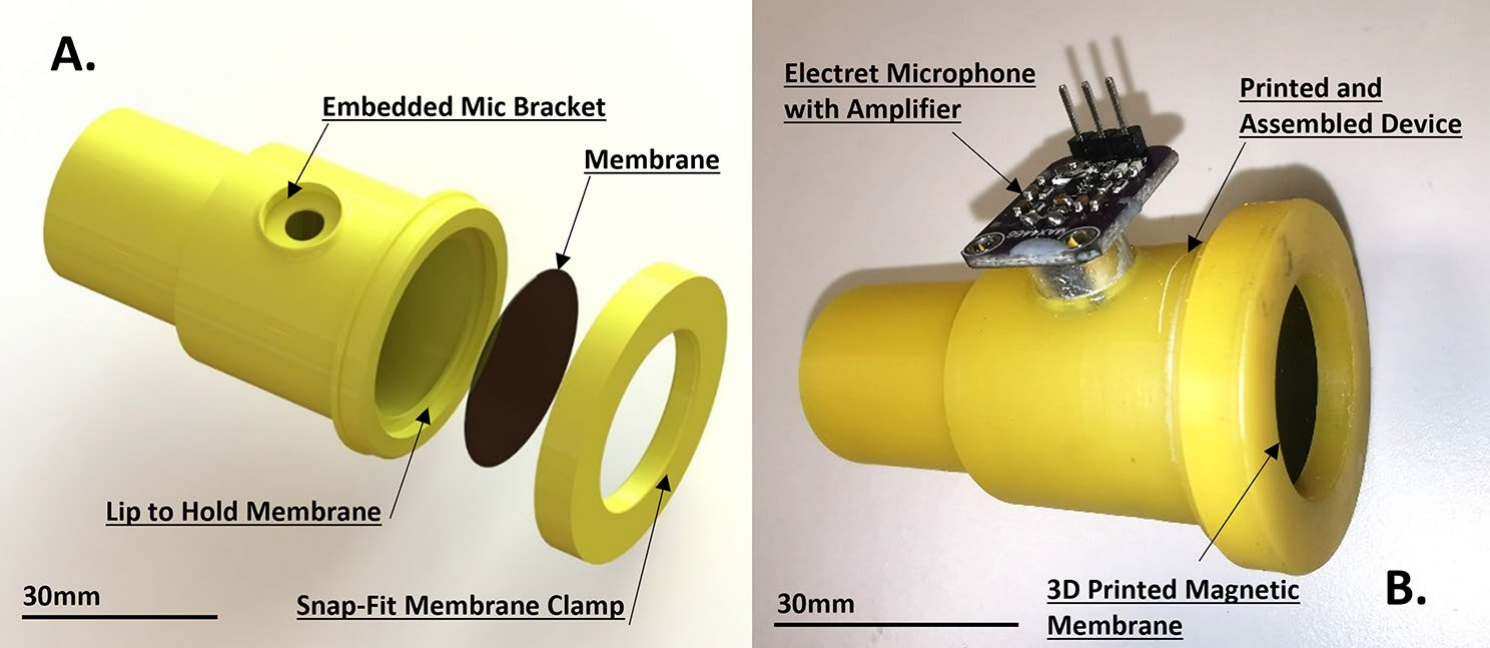
(**A**) Computer-aided design (CAD) file render of the acoustic metamaterial cell components, including the holding support (which includes a bracket to introduce the microphone, allowing for acoustic recordings) and the snap-fit membrane clamp, both 3D-printed using a yellow commercially-available resin, and the magnetic membrane. (**B**) Assembly of the 3D-printed acoustic metamaterial cell, including the electret microphone with an amplifier.

Sub-100 Hz tunability is achieved whilst maintaining ultra-subwavelength dimensions, with total device length $\le \lambda$/77.5. The design, fabrication, and testing of the AMM cell are reported, with novelty in the materials and fabrication processes involved. The magnetic material consisted of a custom-made magnetorheological elastomer formula suitable for SLA 3D-printing, allowing active control of the AMM cell and to be easily and repeatably fabricated. The aim in creating a fully 3D-printed device is to demonstrate the promise additive manufacturing shows to fabricate adjustable metamaterials for broader use. This photo-responsive resin has effectively superparamagnetic properties, meaning that it can align its magnetic dipole moment to the direction of any externally applied magnetic field over 5 mT. This creates a magnetically versatile material that does not require any post-processing poling steps, and displays the final properties *M*, density, and *E* of 2.4 emu/g, 1185.23 kg/m^3^, and 17.89 MPa, respectively. The entirely SLA 3D-printed prototype is shown in Fig. 5. It can be observed from Fig. 4 that the power spectra at resonance for 0 mT applied magnetic field is higher than that measured at 392.5 mT, where one would expect the opposite trend. A possible explanation for this is because of the interference of the applied magnetic field with the electret microphone, leading to a decrease in power.

In summary, this work reports the design, fabrication, testing, and mathematical description of an adjustable acoustic metamaterial cell. This is achieved via SLA 3D-printing of a custom-made magnetic resin that does not require any post processing, making the full process straightforward and repeatable. This work builds on the existing literature of smart acoustic metamaterials by fabricating a device able to tune its operating frequency as a function of an applied magnetic field, establishing an exciting field for the development of the new generation of adaptable devices for low frequency modulation.

## Methods

### Mathematical model

All variables used in the final mathematical model are defined in Supplementary Table S1. The fundamental design of the acoustic metamaterial cell is a Helmholtz resonator coupled to a magnetic membrane. The magnetic membrane is fabricated using a superparamagnetic resin such that, under an applied magnetic field ${\textbf {B}}$, an instantaneous force is applied to the membrane resulting in a tension modulation. To describe this system mathematically, a few assumptions have been taken into consideration: (i) the membrane is thin, isotropic, and homogeneous, and in-plane deformations can be neglected, (ii) the diameter of the membrane must be at least 80 times larger than its thickness, such that the Kirchhoff-Love plate theory can be applied, (iii) membrane vibrations are axisymmetric and harmonic, and (iv) the stress applied to the membrane is uniform. Hence, considering a freely vibrating membrane with uniform pre-stress^24^:

1 $$\begin{aligned} -D\nabla ^4 w+ T\nabla ^2 w=\rho h \frac{\partial ^2 w}{\partial t^2 } \end{aligned}$$

where $\rho$ is the density of the membrane, *w* is its transverse displacement, *T* represents the acting pre-stress on the membrane, and *D* is its flexural rigidity, defined according to Eq. (2)^28^.

2 $$\begin{aligned} D=\frac{E h^3}{12(1-\nu )} \end{aligned}$$

Where *E* and $\nu$ are, respectively, the Young’s modulus and Poisson’s ratio of the membrane. The pre-stress on the membrane can be divided into two elements; mechanical ($T_1$), and magnetic ($T_2$), with *T* being the sum of the two. The magnetic pressure applied to the membrane is defined by Eq. (3)^29^.

3 $$\begin{aligned} P_0=h \mu _0 M\nabla H \end{aligned}$$

where *h* is the thickness of the membrane, $\mu _0$ is the vacuum permeability, *M* is the magnetisation of saturation, and *H* is the strength of the applied magnetic field. When the deflection of the membrane (*z*) is significantly smaller than the radius of the membrane, the mechanical relationships below can be applied^24^.

4 $$\begin{aligned} \varepsilon =(2u^2)/(3a^2 ) \qquad \sigma =(P_0 a^2)/4hu \qquad E=\sigma /\varepsilon \end{aligned}$$

which can be now inserted into the constitutional relations of the system, resulting in Eq. (5).

5 $$\begin{aligned} T_2=\root 3 \of {12D \sigma ^2 \varepsilon }=\left( {\frac{Eh a^2 P_0^2}{24(1-\nu )}} \right) ^{1/3} \end{aligned}$$

Membrane vibrations are assumed to be harmonic and they are described, in polar coordinates, by $u(r,\theta ,t)=\eta _a (r,\theta )e^{i\omega _{n} t}$, such that the vibration mode function is derived from the separation variable method.

6 $$\begin{aligned} u(r,\theta )=[A_n J_n (k_1 r)+B_n I_n (k_2 r)] e^{in\theta } \end{aligned}$$

where *A* and *B* are arbitrary constants derived from the initial conditions, $J_n$ is the $n{th}$ Bessel function of the first kind, $I_n$ is the $n{th}$ solution of the modified first kind, and $k_i$ are the wavenumbers, defined by:

7 $$\begin{aligned} k_{1,2}=\left( \frac{\sqrt{T^2+4D\rho h({\omega _n })^2} {_+^-}T}{2D} \right) ^{1/2} \end{aligned}$$

Considering axisymmetric vibration, and combining the previous expressions, the resonance frequency of the membrane ($\omega _{mem}$) is determined by^30^:

8 $$\begin{aligned} \omega _{mem}=\frac{2.405}{a}\sqrt{\frac{T_1{_-^+}T_2}{\rho h}}=\frac{2.405}{a}\sqrt{\frac{T_1{_-^+}\left( \frac{Eha^2 P_0^2}{24(1-\nu )} \right) ^{1/3} }{\rho h}} \end{aligned}$$

The acoustic behaviour of the membrane-coupled Helmholtz resonator system can be modelled as a mass-spring-damper system. Defining $U_1$ and $U_2$ as, respectively, the volume flow of air displaced at the resonator neck and around the membrane, the governing equations are given by Eqs. (9) and (10), using a 2 degrees of freedom (DOF) system^31^.

9 $$\begin{aligned}&M_1(\dot{U}_1) +K_1\int {(U_1-U_2)}\, dt=P_1 e^{j\omega _F t} \end{aligned}$$

10 $$\begin{aligned}&M_2(\dot{U}_2) +D_2 U_2+K_2 \int {U_2}\,dt+K_1\int {(U_2-U_1)}dt=0 \end{aligned}$$

where *M*, *K*, and *D* are the equivalent mass, stiffness, and damping coefficients. By modelling the system as a piston, these quantities are determined to be^31^:

11 $$\begin{aligned} \begin{array}{llll} M_1=\frac{ \rho _{air} h_{neck}}{S_{neck}} &{} K_1=\frac{\rho _{air} c_{air}^2}{V_{cavity}} &{} \rho _a=\rho _{mem} h\\ M_2=\frac{\mu _n^2 \rho _a}{4\pi a^2 } \qquad &{} K_2=\frac{\mu _n^2 \rho _a \omega _{mem}^2}{4\pi a^2 } \qquad &{} D_2=\frac{\mu _n^2 \rho _a \omega _{mem}^2 \zeta _n}{2\pi a^2 } \qquad &{} \zeta _n=\frac{c_d}{2 \rho _a \omega _{mem}} \end{array} \end{aligned}$$

Where the effective neck of the Helmholtz resonator must be re-defined, due to the viscosity air friction against the resonator walls^32^, as $h_{corrected}=h_{neck}+ 0.6133 r_{neck}$. Finally, to determine the acoustic resonance ($\omega$) of the acoustic metamaterial cell, the model for harmonic oscillation must be considered^33^ and solved, leading to:

12 $$\begin{aligned} \omega =\sqrt{\frac{K_1 M_1+K_1 M_2+K_2 M_1-\sqrt{K_1^2 M_1^2 +2 K_1^2 M_1 M_2+ K_1^2 M_2^2 +2K_1 K_2 M_1^2 -2K_1 K_2 M_1 M_2+ K_2^2 M_1^2 }}{2M_1 M_2 }} \end{aligned}$$

It must be noted that Eq. (12) refers to the resonance frequency of the fundamental acoustic mode. To model higher modes, the 2 DOF system must be expanded (see literature for further detail^31^).

### Synthesis of the magnetic resin and magnetic properties

The SLA 3D-printable magnetic resin was defined by introducing 50–100 nm magnetite (Fe$_3$O$_4$) nanoparticles (3.5$\%$ wt) into a custom-made photo-responsive resin consisting of bisphenol-A ethoxylate diacrylate ($M_{n} \approx$ 1700) as a base monomer, t-octylphenoxypolyethoxyethanol (Triton-X, 10$\%$ wt) as a surfactant, phenylbis (2,4,6 trimethylbenzoyl) phosphine oxide (IRGACURE 819, 2$\%$ wt) as a photoinitiator, and 2,3,5-triphenyltetrazolium chloride (TTZ, 0.1% wt) as a photoblocker. All chemicals were purchased from Merck Life Science UK Limited and used without further modification. Addition of magnetite nanoparticles into the resin was followed by sonication cycles of 1 h at 50 °C to ensure uniform distribution and prevent aggregation. The resin was kept in light-insulating containers while not in use.

A Superconducting Quantum Interference Device (SQUID) magnetometer was used to measure the magnitude and type of magnetisation of the resin. For this measurement, thin samples were 3D printed, weighed, and assembled into an AA size capsule. The weight of the sample and the final recorded magnetisation are used to calculate the magnetisation per gram. The density of the resin formula, without any poling regime, was measured by 3D printing a 10 mm cube and weighing the result. The cube dimensions were re-measured using calipers after printing to account for any printing error. Three samples were used, each with the following regimes and thicknesses: no poling (0.98 ± 0.03 mm); magnetic poling at 150 °C for 2 h (0.64 ± 0.05 mm); and magnetic poling at 300 °C for 2 h (0.79 ± 0.19 mm), respectively. The weights of these samples is shown in Supplementary Table S2.

### Fabrication of the acoustic metamaterial cell

The resonator frame and the membrane were 3D-printed using a PRUSA SL1S (Prusa, Prague, Czech Republic), with the frame being made from the PRUSA yellow tough resin. The frame was 3D-printed with 5 s exposure time per layer, and a burn-in exposure time of 25 s per layer (at 50 microns layer thickness). The magnetic membrane was 3D-printed using the magnetic custom resin, with an exposure time of 60 s per layer, and a burn-in exposure time of 120 s per layer (at 25 microns thickness). The body of the Helmholtz resonator was designed with a small hole in the side to allow for acoustic measurements from the inside of the device via an embedded electret microphone in order to reduce acoustic background noise. The printed part alongside a computer-aided design (CAD) render of the design is shown in Fig. 5.

### Experimental set-up

**Figure 6:**
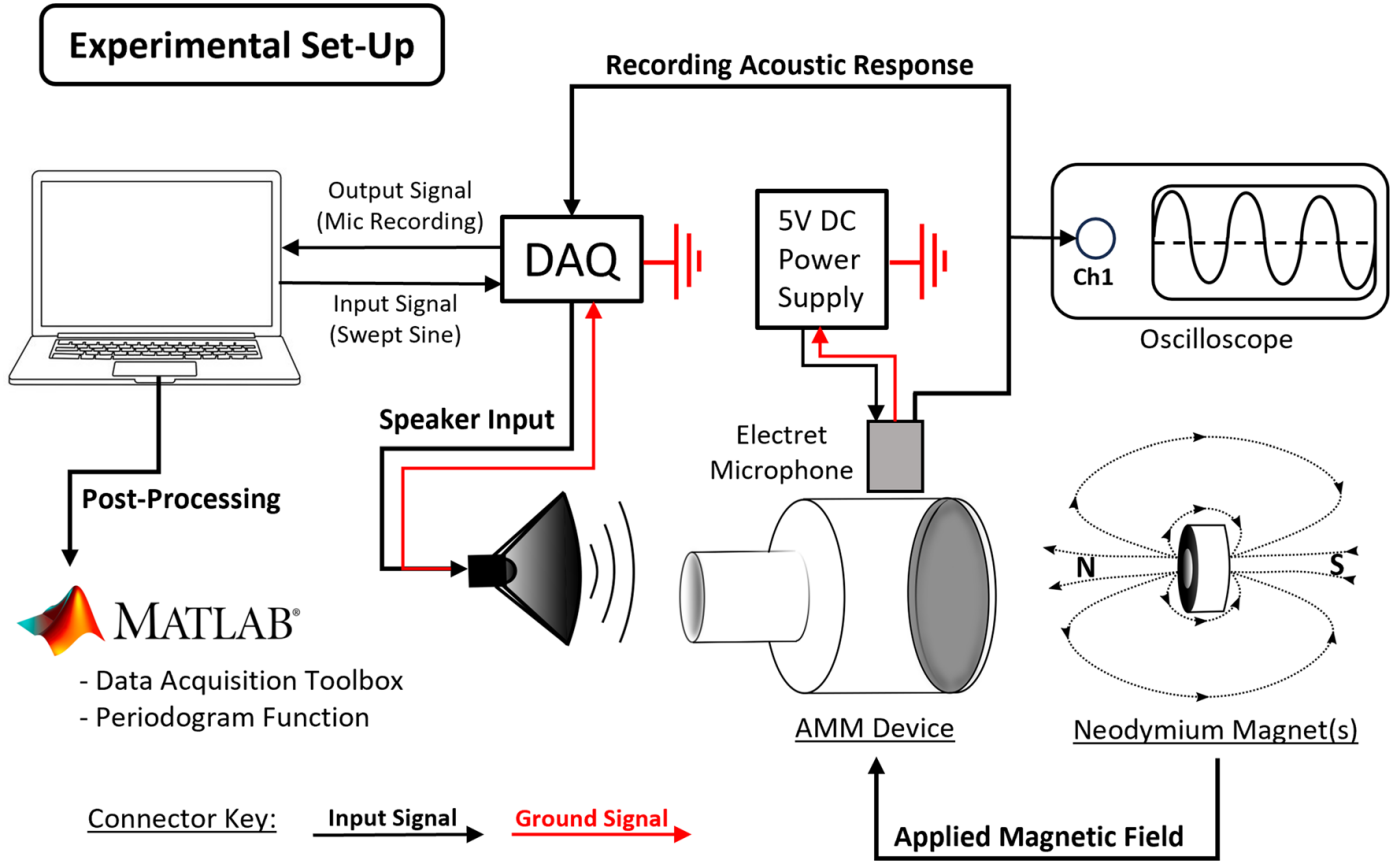
Diagram of the experimental set up used to determine the operation frequency of the 3D-printed acoustic metamaterial cell.

To test the acoustic response of the active acoustic metamaterial cell, a custom rig was designed to accommodate all the requirements of the experiment, as shown in Fig. 6. The components required for this experiment are a National Instruments data acquisition (DAQ) system, an electret microphone, a 5 V power supply, an oscilloscope, a low-frequency speaker, neodymium-iron-boron magnets, and associated fixtures to support the device in place. The DAQ input signal (a sweep from 50 Hz to 400 Hz, 3 V AC amplitude) is generated in MATLAB using the Data Acquisition Toolbox Plug-in and sent to the speaker. The acoustic response of the acoustic metamaterial cell is then recorded using the electret microphone, where the output is fed back into the DAQ, and then into a MATLAB code such that the adequate post processing can be applied. Several acoustic recordings are taken under the application of several magnetic field strengths. This magnetic field is generated by stacking multiple (up to 6) permanent neodymium-iron-boron magnets^34^. The generated magnetic field was measured using an Extech MF100AC/DC Magnetic Field Meter (Extech Instruments)^35^ (details in Supplementary Table S3 and Supplementary Fig. S2).

## Supplementary Information

Supplementary Information.

## Data Availability

The datasets generated during and/or analysed during the current study are available in the Pure data repository at University of Strathclyde, (10.15129/6c664eb1-9ec6-4d71-8751-59526c761aab).
