# Supplementary material for: An adjustable acoustic metamaterial cell using a magnetic membrane for tunable resonance
Source: Sci Rep. 2024 Jul 1;14:15044. doi: 10.1038/s41598-024-65819-2 (PMC11637203; doi:10.1038/s41598-024-65819-2)
Supplement: Supplementary file 1 — Supplementary Information. [file 41598_2024_65819_MOESM1_ESM.pdf]

Supplementary Information - An adjustable acoustic metamaterial cell using a magnetic membrane for tuneable resonance

Alicia Gardiner[1,2,*], Roger Domingo-Roca[2], James F. C. Windmill[2], and Andrew Feeney[1]

[1]Centre for Medical and Industrial Ultrasonics, James Watt School of Engineering, University of Glasgow, Glasgow, G12 8QQ, UK
[2]Centre for Ultrasonic Engineering, Department of Electronic & Electrical Engineering, University of Strathclyde, Glasgow, G1 1XW, UK
[*]a.gardiner.1@research.gla.ac.uk

Mathematical determination of membrane tension and Young's modulus

The tension on the membrane without an applied magnetic field was determined using **Equation S1**[1].

$$T = \left(\frac{2\pi f R}{2.405}\right)^2 \tag{S1}$$

f is the resonance frequency of the membrane (determined experimentally), R is the radius of the membrane, and 2.405 is the first root of the Bessel function of order 0.

The Young's modulus, E, of the membrane was determined using the data recorded with Polytec's MSA-100-3D Micro System Analyzer. A method for determining E of a clamped membrane via it's mechanical resonance frequency has been detailed in the literature[2]. This method exploits the effects of thermoelastic bending on the modulus with the following equation.

$$E = 48\pi^2(1-\nu^2)\rho_m \left(\frac{f}{h}\right)^2 \left(\frac{R}{\lambda_n}\right)^4 \tag{S2}$$

where ν is the Poisson's ratio of the membrane, ρ_m is the density of the membrane, and λ_n is 3,196. To find a comparison for the resin's mechanical qualities, E and ν, the closest equivalents in the literature are related to dental composites. This is because they are often photo-curable and generally contain ceramic inclusions that approximately replicate the size and shape of the magnetic nanoparticles. A study reporting composite biomaterials[3], measured the mechanical properties of bisphenol-A ethoxylate diacrylate with varying percent weights of triethylene glycol dimethacrylate to be 4.3-4.5 GPa, and ν to be 0.334-0.352. Other studies have measured E values of a resin comprising of only crosslinking ethoxylated bisphenol-A dimethacrylates (without composites or other secondary monomers) as 2.16 MPa[4] and 1.134 MPa[5]. Another paper found ν of several different dental fillers with various combinations of monomers and composites (primarily comprised of crosslinking methacrylate/dimethacrylates) to all be within 0.3-0.39[6].

The disparity between values for E is due to the different average molecular weights of BEMA used, along with the different additives, inhibitors, and composites used in these resin variations. ν is more consistent regardless of variations in resin formulation, as such, an informed estimate of ν can be used to inform the mathematical model. A membrane clamp that replicates the mechanical pre-stress applied by the device was fabricated and assembled with an identical membrane to that used in the experimental data collection. The membrane's resonant properties were measured using a Micro System Anayzer (MSA-100-3D, Polytech, GmbH), with a layer of foam underneath to closely mimic free boundary conditions (**Supplementary Figure S1**. A full scan of the membrane's surface was conducted with a frequency resolution of 0.3125 Hz.

DAQ parameters and data post-processing

The DAQ parameters for generating the input signal and recording the output signal are as follows: sampling frequency is 25,000 points per second, the AC input voltage is 3 V, start chirp frequency is 50 Hz, end chirp frequency is 150 Hz, total run-time per pass is 300 seconds, and total number of data points per pass is 7,500,000. Several passes were recorded to build up a robust data set, so the total number of data points is a product of the number of points per pass and the number of magnetic testing conditions (see **Supplementary Table S3**).

The obtained acoustic recordings were processed in MATLAB using the following steps:

- Load system variables and data sets.
- Remove the effect of the microphone amplifier by setting each recording's baseline to zero.
- Use '*periodogram*' with a rectangular window size of the dataset to convert the signal to the frequency domain and find the power spectra of each recording.
- Identify the peak power and find the corresponding resonance frequency.
- Compare recordings by plotting the power (dB) against the frequency bandwidth (Hz).

Supplementary Table S1 - Definitions

Definitions and Values used in the Mathematical Model			
Variable	Symbol	Value	Units
Density of Air	ρ_{air}	1.293[7]	Kg/m^3
Speed of Sound in Air	c_{air}	344[8]	m/s
Height of Helmholtz Resonator Neck	h_{neck}	2.5×10^{-3}	m
Area of Neck Opening	S_{neck}	3.1416×10^{-4}	m
Volume of HR Cavity	V_{cavity}	1.3273×10^{-5}	m^3
Density of Membrane	ρ_{mem}	1185.23	Kg/m^3
Thickness of Membrane	h	2.6×10^{-4}	m
Nth solution to the Bessel function of the 1st kind, 0th order ($n = 1$)	μ_n	2.405[1]	-
Membrane Radius	a	13×10^{-3}	m
Fundamental/Natural Frequency of Membrane	ω_{mem}	-	$Rads/s$
Damping Ratio of Membrane	ζ_n	0.005	-
Viscous Damping Coefficient	c_d	-	Ns/m
Volume Velocity of HR air at Neck	U_1	-	m^3/s
Volume Velocity of air around Membrane for 1st mode	U_2	-	m^3/s
Normal Displacement of Membrane	z	-	m
Time Elapsed	t	-	s
Pressure of Acoustic Excitation	P_1	0	N
Frequency of Excitation	ω_F	-	$Rads/s$
Acoustic Fundamental Resonant Frequency of Device	ω	-	$Rads/s$
Flexural Stiffness of membrane	D	-	Nm
Tension per unit Length	T	-	N/m
Mechanical Pre-stress	T_1		N/m

Definitions and Values used in the Mathematical Model			
Variable	Symbol	Value	Units
Magnetic Pre-stress	T_2	-	N/m
Young's Modulus of Membrane	E	17.89×10^6	N/m^2
Poisson's Ratio of Membrane	υ	0.35	-
Magnetisation of Saturation per unit Volume	M	2.8446×10^3	A/m^2
Applied Magnetic Field Strength	H	-	A/m
Vacuum Permeability	μ_0	$4\pi \times 10^{-7}$[9]	N/A^2
Magnetic body Force on Membrane (Normal force per unit area)	P_0	-	N/m^2

Supplementary Table S2 - Magnetic Sample Specifications

Custom Resin Samples (100-50nm Fe3O4, 3.5%wt)			
Poling Regime	Density (Kg/m^3)	Mass Capsule **(g)**	Mass Sample **(g)**
None	1185.23	0.041	0.023
150°C, 2hrs	1185.23	0.041	0.0165
300°C, 2hrs	1185.23	0.041	0.023

Supplementary Table S3 - Measured magnetic field from each array of magnets tested in this experiment.

No. Magnets	**0**	**1**	**2**	**3**	**4**	**5**	**6**
Maximum Magnetic Field **(mT)**	0	160	263	321	356	399	422
Minimum Magnetic Field **(mT)**	0	85	151	240	273	301	363
Average Magnetic Field **(mT)**	0	122.5	207	280.5	314.5	350	392.5

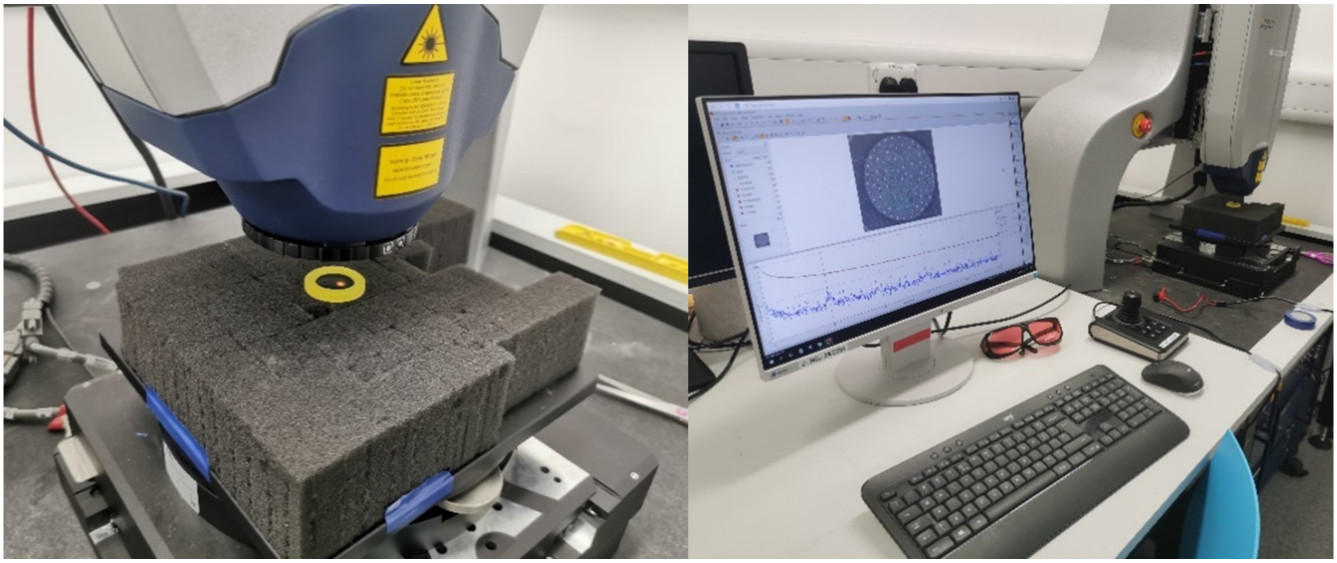

Supplementary Figure S1 - Clamped membrane undergoing measurements to capture the 1st resonant mode in Polytec's MSA-100-3D Micro System Analyzer.

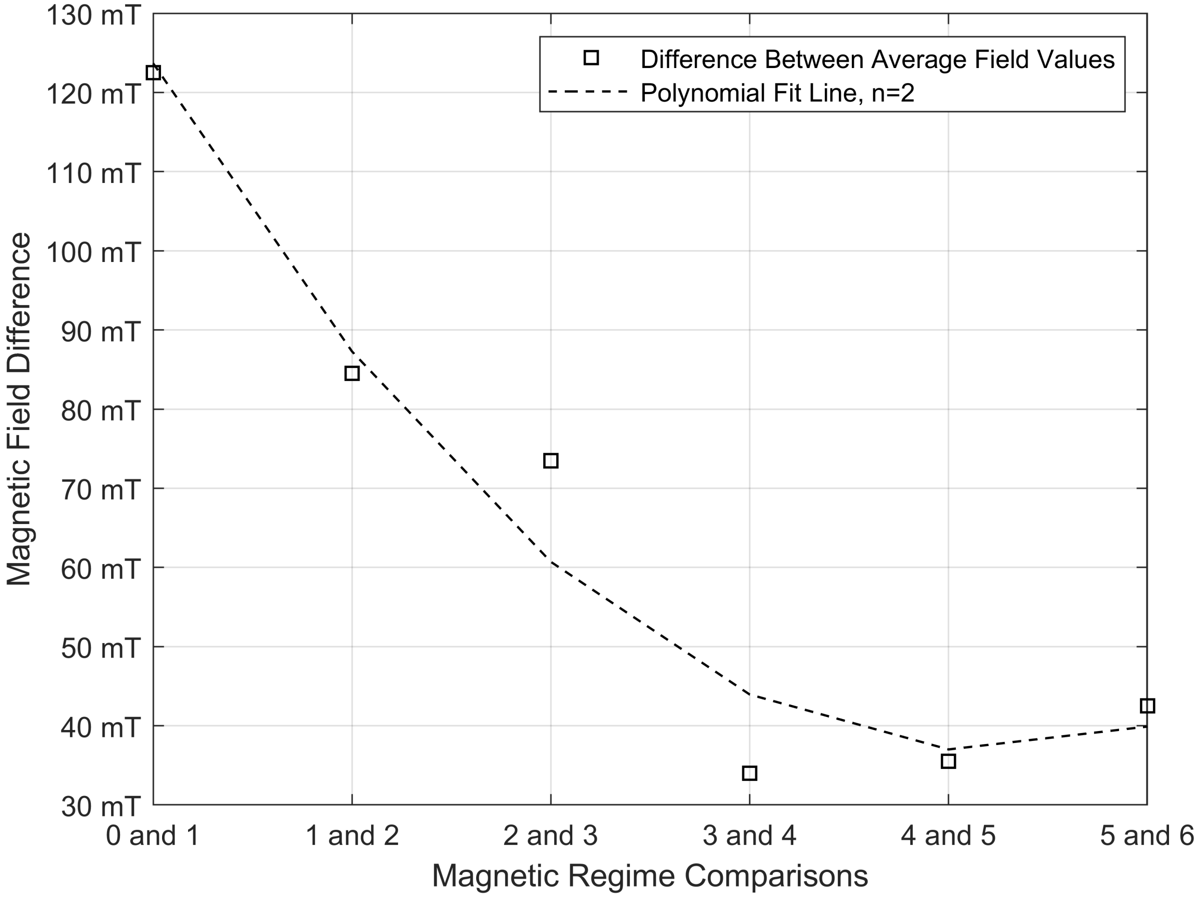

Supplementary Figure S2 - Difference in magnetic field between each adjacent magnet stacking regime used experimentally. Regimes are detailed in Supplementary Table S3

References

1. Leissa, A. Membranes. In Braun, S. (ed.) *Encyclopedia of Vibration*, 762–770, DOI: https://doi.org/10.1006/rwvb.2001.0132 (Elsevier, Oxford, 2001).

2. Daniel Macias, J., Ordonez-Miranda, J. & Alvarado-Gil, J. J. Resonance frequencies and Young's modulus determination of magnetorheological elastomers using the photoacoustic technique. *J. Appl. Phys.* **112**, 124910, DOI: 10.1063/1.4770473 (2012). https://pubs.aip.org/aip/jap/article-pdf/doi/10.1063/1.4770473/15103039/124910_1_online.pdf.

3. Jones, D. W. & Rizkalla, A. S. Characterization of experimental composite biomaterials. *J. Biomed. Mater. Res.* **33**, 89–100, DOI: https://doi.org/10.1002/(SICI)1097-4636(199622)33:2<89::AID-JBM5>3.0.CO;2-H (1996).

4. Moraes, R. R. *et al.* Preparation and evaluation of dental resin luting agents with increasing content of bisphenol-a ethoxylated dimethacrylate. *J. Biomater. Appl.* **24**, 453–473, DOI: 10.1177/0885328208098594 (2010).

5. Sideridou, I., Tserki, V. & Papanastasiou, G. Study of water sorption, solubility and modulus of elasticity of light-cured dimethacrylate-based dental resins. *Biomaterials* **24**, 655–665, DOI: https://doi.org/10.1016/S0142-9612(02)00380-0 (2003).

6. Chung, S. M., Yap, A. U. J., Koh, W. K., Tsai, K. T. & Lim, C. T. Measurement of poisson's ratio of dental composite restorative materials. *Biomaterials* **25**, 2455–2460, DOI: 10.1016/j.biomaterials.2003.09.029 (2004).

7. N.A.S.A. Earth science data systems - "air mass/density" (2023). https://www.earthdata.nasa.gov/topics/atmosphere/atmospheric-pressure/air-mass-density [Accessed on (07/12/2023)].

8. Truax, B. Speed of sound (2023). https://www.sfu.ca/sonic-studio-webdav/handbook/Speed__Of_Sound.html [Accessed on (07/12/2023)].

9. Goldfarb, R. B. The permeability of vacuum and the revised international system of units. *IEEE Magn. Lett.* **8**, 1–3, DOI: 10.1109/LMAG.2017.2777782 (2017).
